# Development and analysis of long non-coding RNA-associated competing endogenous RNA network for osteosarcoma metastasis

**DOI:** 10.1186/s41065-021-00174-0

**Published:** 2021-02-16

**Authors:** Yucheng Fu, Qi Liu, Qiyuan Bao, Junxiang Wen, Zhuochao Liu, Yuehao Hu, Guoyu He, Cheng Peng, Yiqi Xu, Weibin Zhang

**Affiliations:** Department of Orthopedics, Ruijin Hospital, Shanghai Jiaotong University School of Medicine, 197 Ruijin Er Road, Shanghai, 200025 PR China

**Keywords:** Osteosarcoma, Metastasis, Competing endogenous RNA, Bioinformatic analysis, Long non-coding RNA

## Abstract

**Background:**

Osteosarcoma is the primary bone malignant neoplasm that often develops metastasis. Increasing evidences have shown that non-coding RNAs (ncRNAs) relate to the progression of osteosarcoma. However, the ncRNAs’ roles in osteosarcoma metastasis are still unknown.

**Methods:**

Differentially expressed (DE) RNAs were identified from Gene Expression Omnibus (GEO) database. Protein-protein interaction (PPI) of DE messenger RNAs (DEmRNAs) was built through STRING database. The target mRNAs and long ncRNAs (lncRNAs) of microRNAs (miRNA) were predicted through miRDB, Targetscan and Genecode databases, which then cross-checked with previously obtained DERNAs to construct competing endogenous RNA (ceRNA) network. All networks were visualized via Cytoscape and the hub RNAs were screened out through Cytoscape plug-in Cytohubba. The gene functional and pathway analyses were performed through DAVID and MirPath databases. The survival analyses of hub RNAs were obtained through Kaplan-Meier (KM) survival curves.

**Results:**

Five hundred sixty-four DEmRNAs, 16 DElncRNAs and 22 DEmiRNAs were screened out. GO functional and KEGG pathway analyses showed that DERNAs were significantly associated with tumor metastasis. The ceRNA network including 6 lncRNAs, 55 mRNAs and 20 miRNAs were constructed and the top 10 hub RNAs were obtained. Above all, PI3K/AKT signaling pathway was identified as the most important osteosarcoma metastasis-associated pathway and its hub ceRNA module was constructed. The survival analyses showed that the RNAs in hub ceRNA module closely related to osteosarcoma patients’ prognosis.

**Conclusions:**

The current study provided a new perspective on osteosarcoma metastasis. More importantly, the RNAs in hub ceRNA module might act as the novel therapeutic targets and prognostic factors for osteosarcoma patients.

**Supplementary Information:**

The online version contains supplementary material available at 10.1186/s41065-021-00174-0.

## Introduction

Osteosarcoma, one of the most common primary musculoskeletal system malignant tumors, often affects children and young adults [1]. With the development of multidiscipline therapy, especially complete resection of tumor with multi-agent chemotherapy, the 5-year survival rate of patient has significantly improved to over 70% [2]. However, a large number of patients who receive standard or aggressive treatment still develop metastasis and their 5-year survival rates dramatically decrease to less than 20% [3, 4]. Therefore, the promising strategy to improve the prognosis of metastasis patients has been widely investigated. Unfortunately, little progression has been acquired since the underlying mechanisms of invasion and metastasis are still unclear [5].

Non-coding RNAs (ncRNAs) are a group of RNAs that have no protein-coding functions and play important roles in cellular physiology and pathology. Based on the length of nucleotide, ncRNAs are divided into small ncRNAs (< 200 bp) and long ncRNAs (200 to 100 kb). MicroRNAs (miRNAs), one of the most widely studied small ncRNAs, are associated with cell proliferation, differentiation, apoptosis [6]. After gene transcription, miRNAs could bind to 3’UTR (untranslated regions) of targeted messenger RNAs (mRNAs) and prevent their translation, which play vital roles in tumor occurrence and metastasis [7]. Unlike miRNAs, long ncRNAs (lncRNAs) take part in epigenetic, transcriptional, post-transcriptional and translational processes in various cells [8]. A large number of studies have revealed that lncRNAs correlate with tumor progression and act as the biomarkers for diagnosis and prognosis. Some researches even find lncRNAs would be optimistic therapeutic targets of cancers [9–13].

The competing endogenous RNA (ceRNA) hypothesis is proposed as a novel regulatory mechanism between coding RNAs and ncRNAs. Both lncRNAs and miRNAs are important components in this network. LncRNAs can bind to miRNAs through miRNA-binding sites (MREs) and act as miRNA sponges to regulate mRNA expression [14]. Plenty of researchers have constructed different ceRNA networks in various cancers. Lei et al analyzed the data from The Cancer Genome Atlas (TCGA) database and built up a lncRNA-miRNA-mRNA regulatory network in muscle-invasive bladder cancer [15]. Fang et al constructed a ceRNA network of head and neck squamous cell carcinoma from 546 patients’ profiles [16]. However, studies of ceRNA networks in osteosarcoma patients are limited and the role of lncRNA-miRNA-mRNA regulatory network between metastatic and non-metastatic osteosarcoma is still unclear.

In the present study, we obtained the differentially expressed (DE) miRNAs, mRNAs and lncRNAs between metastatic and non-metastatic patients from GSE39040 and GSE39055 datasets. The online databases was used to predict target mRNAs and lncRNAs of miRNAs. Then the ceRNA regulatory network was constructed. We also performed Gene Ontology (GO), Kyoto Encyclopedia of Genes and Genomes (KEGG) pathway and protein-protein interaction (PPI) network analyses of DEmiRNAs and DEmRNAs. Moreover, we identified the osteosarcoma metastasis-associated hub ceRNA module based on the KEGG pathway analysis. Kaplan-Meier (KM) survival analysis was conducted to verify the relationships between hub ceRNAs and prognosis. We hope that the present study would extend our knowledge of osteosarcoma metastasis-associated ceRNA network and provide potential therapeutic targets and prognostic markers for osteosarcoma patients.

## Materials and methods

### Microarray dataset selection

The expression profiles of osteosarcoma were searched from the Gene Expression Omnibus (GEO, http://www.ncbi.nlm.nih.gov/geo). The microarray datasets involved in this study should meet the following criteria: (1) the tumors were confirmed as osteosarcoma by histology; (2) the datasets contained metastatic and non-metastatic patients; (3) the datasets had complete prognosis-associated information; (4) the sample size in the dataset was more 10. Finally, two GEO datasets were selected for further study. The miRNA data were downloaded from GSE39040(platform GPL15762, Illumina Human v2 MicroRNA Expression BeadChip, Illumina, Inc., San Diego, CA, USA) which contained 65 osteosarcoma patients. The mRNA and lncRNA data were downloaded from GSE39055(platform GPL14951, Illumina HumanHT-12 WG-DASL V4.0 R2 Expression BeadChip, Illumina, Inc., San Diego, CA, USA) which had 37 osteosarcoma patients. The clinical characteristics of patients in GSE39040 and GSE39055 were shown in Supplementary Table 1.

### Differential expression analysis

**Fig. 1:**
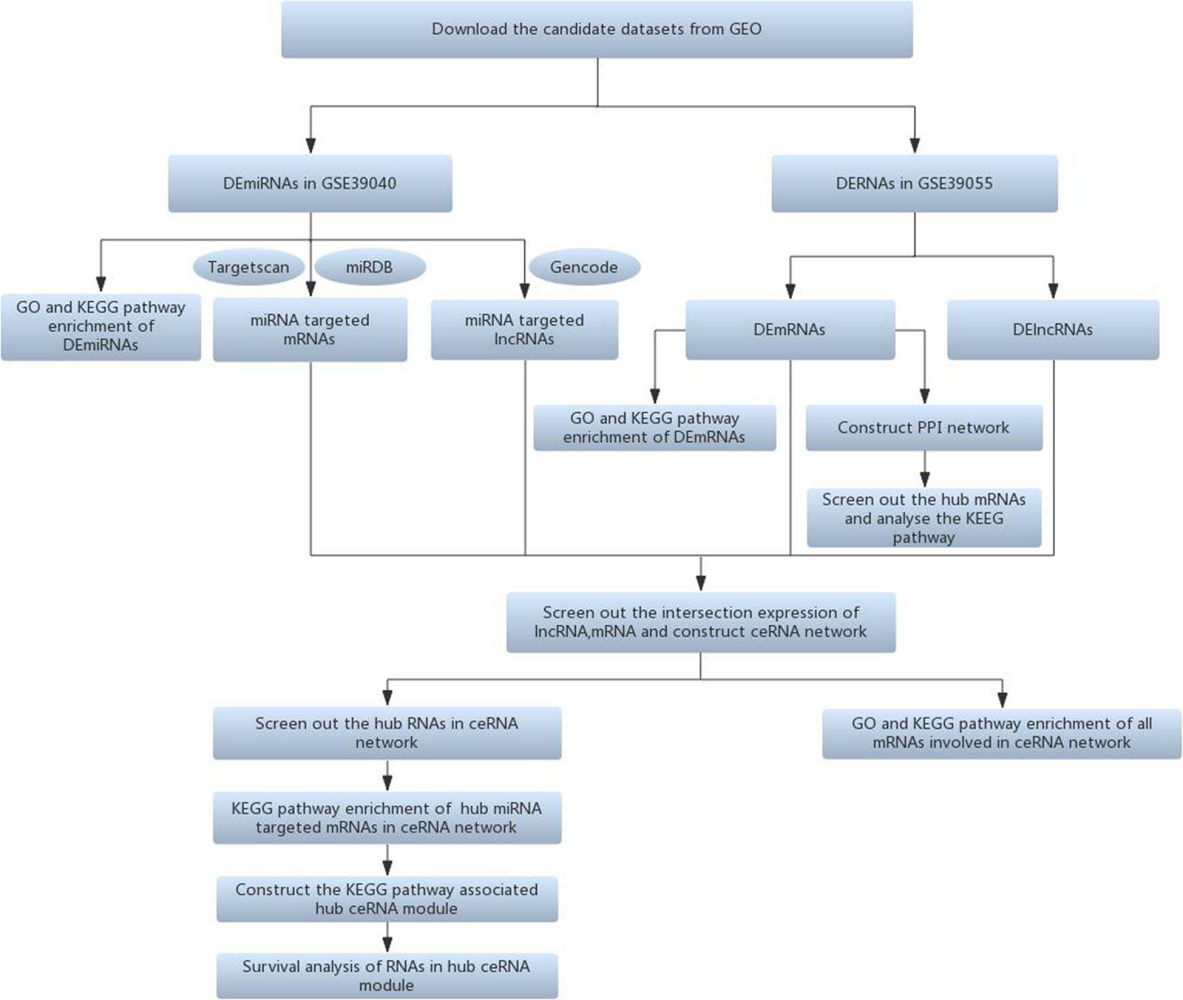
Flow chart of the study. GO:gene ontology; DEmRNA:differentially expressed messenger RNA; DEmiRNA:differentially expressed microRNA; DElncRNA:differentially expressed long non-coding RNA; KEGG:Kyoto Encyclopedia of Genes and Genomes; ceRNA:competing endogenous RNA; PPI:Protein-protein interaction

The probe names in the microarray datasets were converted into RNA names and multiple values of the same gene were averaged by R software (version 3.61). Patients from selected datasets were divided into metastatic and non-metastatic groups. The Linear Models for Microarray Analysis (Limma) package was applied to perform DERNAs analysis between two groups. The cutoff value of DERNAs was P<0.05 and |fold change (FC)|>1.5. The heat map was drawn through ‘pheatmap’ package in R software. According to the Gene database information (https://www.ncbi.nlm.nih.gov/gene) and classification of RefSeq accession numbers and molecule types (https://www.ncbi.nlm.nih.gov/books/NBK21091/table/ch18.T.refseq_accession_numbers_and_mole/?report=objectonly), the DERNAs in GSE39055 were divided into DEmRNA (‘NM_’ or ‘XM_’) and DElncRNA (‘NR_’ or ‘XR_’) groups. The flow chart of this study was shown in Fig. 1.

### Prediction of miRNA targets

The target mRNAs of DEmiRNA were screened from Targetscan (http://www.targetscan.org/vert_72) and miRDB (http://mirdb.org/) respectively [17, 18]. Then the intersectional mRNAs in two databases were obtained for further study. The target lncRNAs of DEmiRNA were selected from Genecode (https://www.gencodegenes.org/) by overlapping miRNA’s seed region to lncRNAs.

### Functional and pathway enrichment analyses

GO functional and KEGG pathway enrichment analyses of all DEmRNAs and mRNAs in ceRNA network were performed by Database for Annotation, Visualization and Integrated Discovery (DAVID, https://david.ncifcrf.gov/) [19]. GO functional enrichment analysis included cellular component (CC), molecular function (MF), and biological process (BP) [20]. MirPath (http://snf-515788.vm.okeanos.grnet.gr) was used to elucidate the GO functional and KEGG pathway enrichment of DEmiRNAs [21]. *P*-value<0.05 was defined as significantly enriched.

### Protein-protein interaction (PPI) network construction and analysis

To study the interaction of all DEmRNAs, the online tool Search Tool for the Retrieval of Interacting Genes database (STRING, https://string-db.org/) was used to construct PPI network [22]. The cutoff confidence score was 0.4 and the disconnected nodes were hid in the network. Then the network was visualized in Cytoscape(v3.7.1) and the top 10 hub mRNAs were screened out by Cytoscape plug-in CytoHubba. KEGG pathway analysis of hub mRNAs was also conducted by DAVID and *p*-value<0.05 was defined as significantly enriched.

### ceRNA network construction and analysis

The overlapping mRNAs were obtained from the intersection of DEmRNAs from GSE39055 and target mRNAs of DEmiRNA. The same method was used to identify overlapping lncRNAs. Then the inverse expression pairs of miRNA-mRNA and miRNA-lncRNA were selected to construct ceRNA network via Cytoscape. Top 10 hub RNAs in ceRNA network were also screened out by CytoHubba. The KEGG pathway analysis of miRNAs in hub RNAs was conducted through their targeted mRNAs in ceRNA network by DAVID. *P*-value<0.05 was defined as significantly enriched. Moreover, the most important osteosarcoma metastasis-associated hub ceRNA module was identified and constructed through KEGG pathway analysis results. The hub ceRNA module was visualized by Cytoscape.

### Survival analysis

The prognostic information of osteosarcoma patients was obtained from the selected dataset. All Patients were divided into high- and low-expression groups based on the expression level of RNAs in hub ceRNA module. The Kaplan-Meier survival analysis of RNAs in hub ceRNA module was conducted by ‘survival’ package in R software. *P <* 0.05 was considered as statistically significant.

## Results

### Identification of DEmiRNAs, DEmRNAs and DElncRNAs

**Fig. 2:**
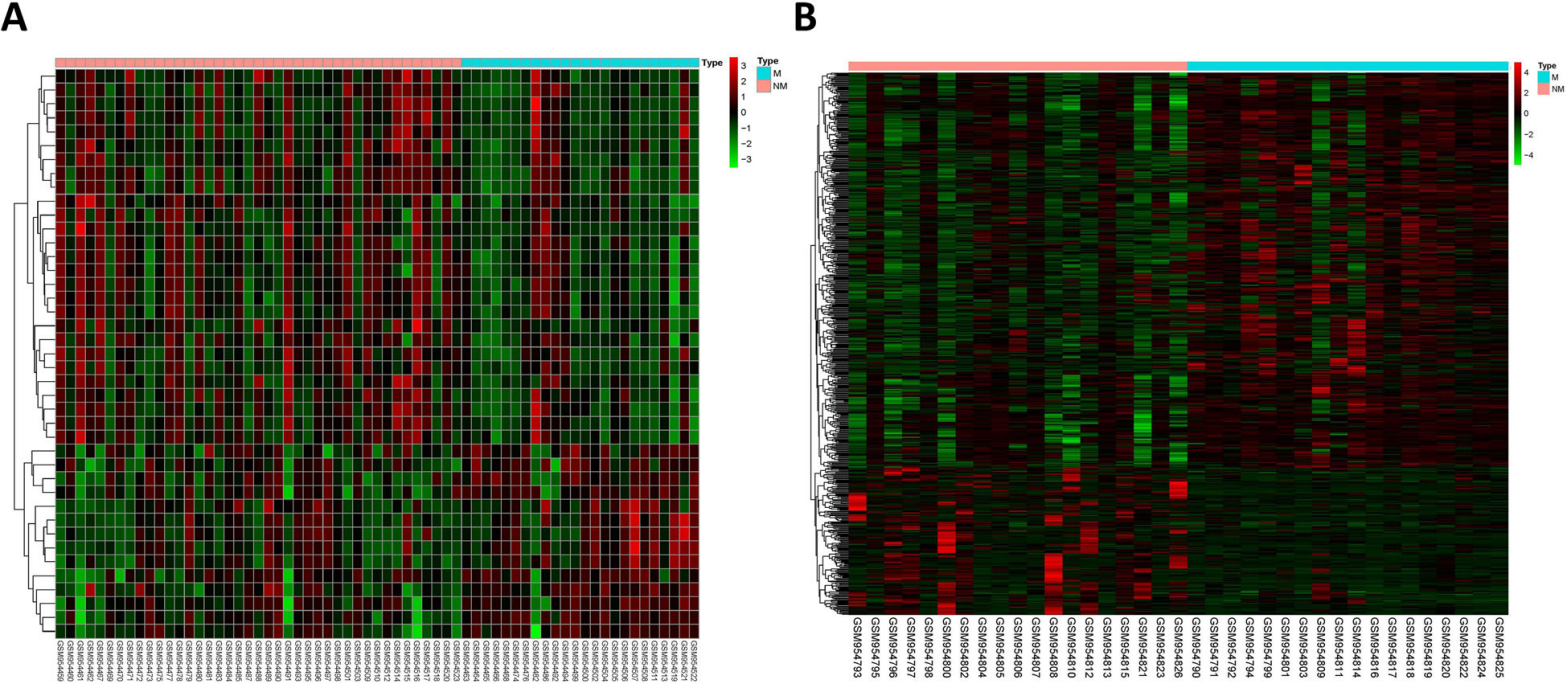
The heat map of DEmiRNAs in GES39040(**a**) and DERNAs in GES39055(**b**). Red and greed indicated up and down-regulated genes between metastasis and non-metastasis groups. M:metastasis; NM: non-metastasis; FC:fold change; DEmiRNA:differentially expressed microRNA

The miRNA expression data were obtained from the GSE39040 dataset, which contained 24 metastatic and 41 non-metastatic osteosarcoma patients. A total of 41 DEmiRNAs were found and the distribution characteristics of these genes were shown in Fig. 2a. 22 of 41 DEmiRNAs IDs could convert into MiRBase IDs for further study and the information of these DEmiRNAs were shown in Table 1.

**Table 1:** The information of all selected DEmiRNAs in GSE39040

miRNA ID	MiRBase ID	Regulation	Log_**2**_(FC)	***P*** value
hsa-miR-324-5p	hsa-miR-324-5p	down	−0.82278253	0.004441989
hsa-miR-1268	hsa-miR-1268a	down	−0.807378661	0.012636846
hsa-miR-202*:9.1	hsa-miR-202-5p	down	−0.796046965	0.044198514
hsa-miR-638	hsa-miR-638	down	−0.786812097	0.037777589
hsa-miR-1183	hsa-miR-1183	down	−0.781217092	0.003659417
hsa-miR-885-3p	hsa-miR-885-3p	down	−0.67843564	0.041117786
hsa-miR-612	hsa-miR-612	down	−0.646006747	0.005810682
hsa-miR-1304	hsa-miR-1304-5p	down	−0.631698619	0.008422893
hsa-miR-296-3p	hsa-miR-296-3p	down	−0.619209613	0.001821016
hsa-miR-99b	hsa-miR-99b-3p	up	0.593231012	0.012834992
hsa-miR-218	hsa-miR-218-5p	up	0.597963257	0.016372671
hsa-miR-10a	hsa-miR-10a-5p	up	0.600685263	0.014459757
hsa-miR-337-3p	hsa-miR-337-3p	up	0.60102361	0.037744275
hsa-miR-432	hsa-miR-432-3p	up	0.611569268	0.018705875
hsa-miR-342-3p	hsa-miR-342-3p	up	0.620361269	0.003592927
hsa-miR-139-5p	hsa-miR-139-5p	up	0.63919661	0.003626249
hsa-miR-495	hsa-miR-495-3p	up	0.642715011	0.000707477
hsa-miR-664	hsa-miR-664a-5p	up	0.665366105	0.005287393
hsa-miR-10b	hsa-miR-10b-5p	up	0.705213072	0.000648507
hsa-miR-329	hsa-miR-329-3p	up	0.710318117	0.007137255
hsa-miR-148b	hsa-miR-148b-3p	up	0.788851193	0.010753646
hsa-miR-487b	hsa-miR-487b-3p	up	0.880742626	0.001741188

*DEmiRNA* differentially expressed microRNA; *FC*fold change

The GSE39055 dataset, which contained 19 metastatic and 18 non-metastatic osteosarcoma patients, was used to analyze DEmRNAs and DElncRNAs. A total of 600 DERNAs were identified and the heat map was used to visualize the different expression values (Fig. 2b). Five hundred sixty-four demrnas and 16 DElncRNAs in these DERNAs were identified for further study. The top 10 up and down-regulated DEmRNAs and all DElncRNAs were listed in Tables 2 and 3.

**Table 2:** The information of top 10 up and down regulation DEmRNAs in GES39055

mRNA symbol	Regulation	Log_**2**_(FC)	***P*** value
PAX5	down	−1.306041775	0.034352544
FAM179A	down	−1.31104379	0.048817304
TEN1	down	−1.316087389	0.018927464
HSD17B3	down	−1.338280872	0.023832893
LOC648148	down	−1.355176906	0.023126387
DCAF7	down	−1.361960103	0.032984616
MRPL9	down	−1.534675909	0.010487122
GAGE12C	down	−1.58044573	0.030207474
GNG10	down	−1.704725672	0.01285595
GAGE12B	down	−1.769143614	0.013737857
FLJ42957	up	1.377523174	0.02310915
RCN3	up	1.38421278	0.005795941
ZMYM1	up	1.453911131	0.000816223
PLD2	up	1.470713457	0.00040628
FLJ36070	up	1.497727783	0.018892588
KIF1A	up	1.568833787	0.013658482
GPR124	up	1.579761576	0.01799364
CCND3	up	1.636336346	0.001881521
FAM116B	up	1.731701273	0.000560124
AKAP8L	up	1.84062885	0.000146584

*DEmRNA* differentially expressed messenger RNA; *FC* fold change

**Table 3:** The information of all DElncRNAs inGES39055

lncRNA symbol	Regulation	Log_**2**_(FC)	***P*** value
OR6W1P	down	−0.783471763	0.042500183
LBX2-AS1	down	−0.790202768	0.045540948
EP400NL	down	−0.825219671	0.004375387
KMT2E-AS1	up	0.713029339	0.006163702
LINC00323	up	0.737302724	0.027993039
MIR137HG	up	0.786870116	0.039509415
SCARNA18	up	0.832769899	0.037372141
SCARNA17	up	0.848340468	0.031726142
TPT1-AS1	up	0.93101518	0.012854532
HSP90B3P	up	1.020964547	0.026633407
RMRP	up	1.040278844	0.049484991
SCARNA11	up	1.129354127	0.0277887
PP7080	up	1.21696745	0.004365053
SCARNA6	up	1.284599057	0.00957118
ZNF252P	up	1.28633713	0.01880755
MRPL20-AS1	up	1.369272878	0.000821295

*DElncRNA* differentially expressed long non-coding RNA; *FC* fold change

### GO functional and KEGG pathway analyses of DEmiRNAs and DEmRNAs

**Fig. 3:**
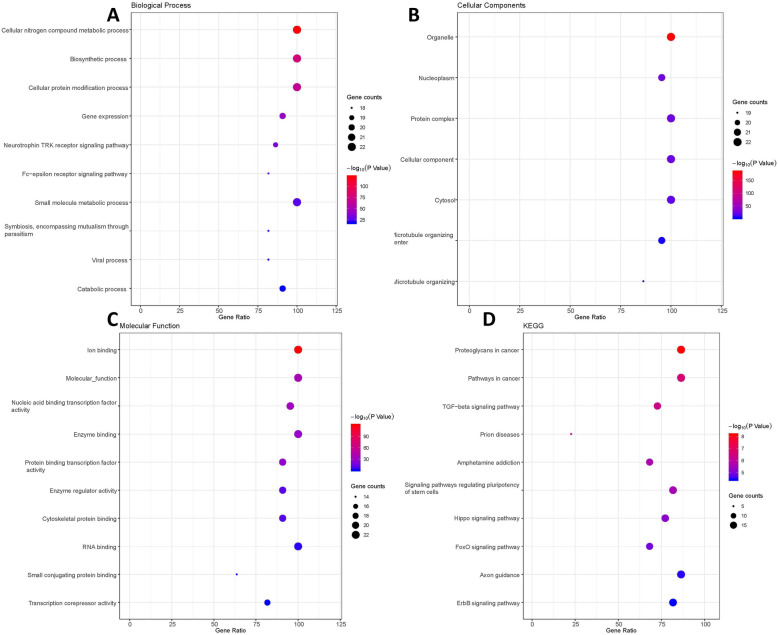
GO functional and KEGG pathway analyses of DEmiRNA. **a** top 10 significantly enriched GO biology process; **b**7 significantly enriched GO cellular components; **c** top 10 significantly enriched molecular function; **d** top 10 significantly enriched KEGG pathways. GO:gene ontology;KEGG:Kyoto Encyclopedia of Genes and Genomes;DEmRNA: differentially expressed messenger RNA

In order to understand the potential biological functions of these DEmiRNAs, GO functional and KEGG pathway analyses were conducted by MirPath. In BP ontology, the DEmiRNAs were involved in several tumor metastatic processes such as biosynthetic process, cellular protein modification process, gene expression and catabolic process (Fig. 3a). As for CC analysis, the DEmiRNAs were mainly distributed in organelle, nucleoplasm, protein complex, cytosol, microtubule organizing center (Fig. 3b). In MF category, DEmiRNAs were encriched in ion binding, nucleic acid binding transcription factor activity, enzyme binding, protein binding transcription factor activity, enzyme regulator activity, cytoskeletal protein binding, RNA binding and transcription corepressor activity (Fig. 3c). Most of these functions took part in tumor metastasis processes through regulating gene expression, protein binding or enzyme activity [23–25].

In addition, the KEGG pathway analysis indicated that DEmiRNAs enriched in 53 different pathways (Supplementary Table 2) and the top 10 pathways were shown in Fig. 3d. A large amount of common tumor metastasis-related pathways such as TGF-beta signaling pathway, Hippo signaling pathway, Ras signaling pathway, Wnt signaling pathway and PI3K/Akt signaling pathway were involved.

**Fig. 4:**
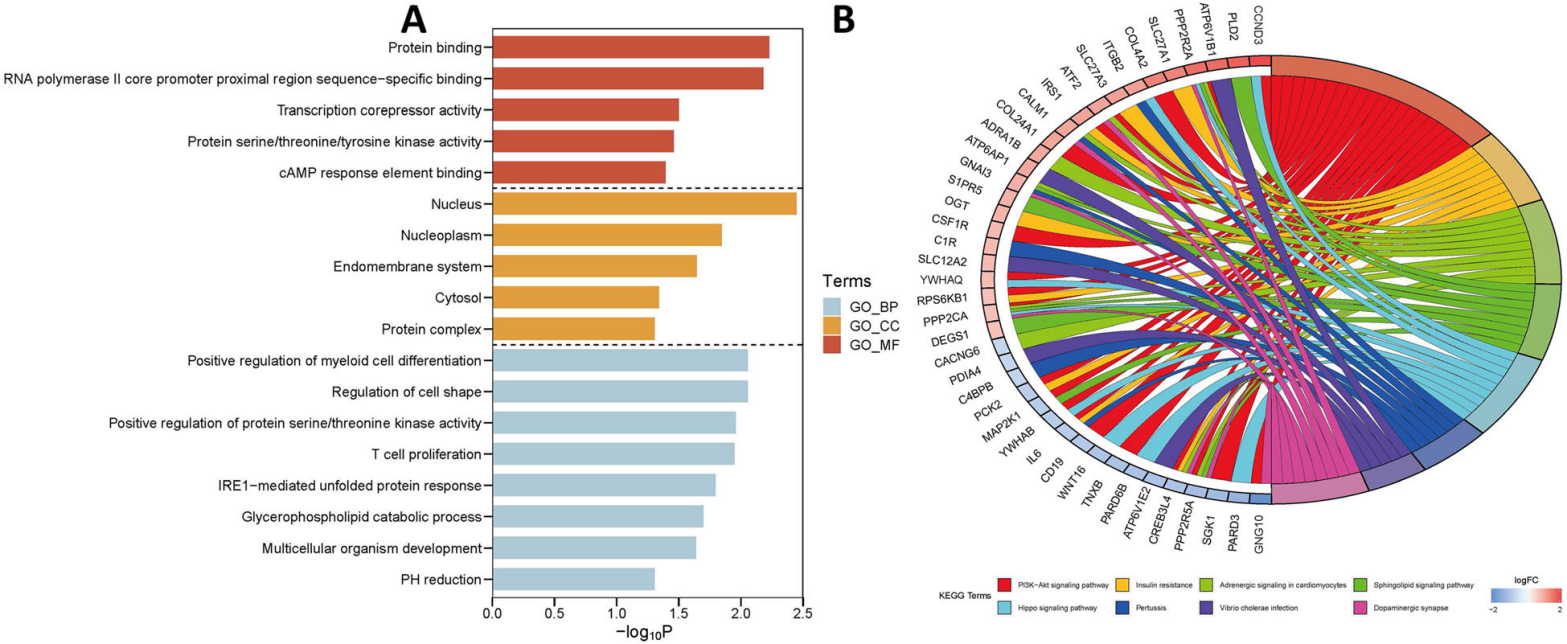
GO functional and KEGG pathway analyses of DEmRNA in GSE39055. **a** significantly enriched GO items in CC, BP and MF; **b** significantly enriched KEGG pathways. GO:gene ontology; KEGG:Kyoto Encyclopedia of Genes and Genomes; FC:fold change; DEmRNA: differentially expressed messenger RNA;BP: biology process;CC:cellular component; MF: molecular function

The online tool DAVID was used to perform GO functional and KEGG pathway analysis for DEmRNAs. The significant result of GO functional enrichment was shown in Fig. 4a. Plenty of items, such as transcription corepressor activity, cAMP response element binding, protein serine/threonine/tyrosine kinase activity were closely related to the process of tumor metastasis [26–28].

KEGG pathway analysis showed that DEmRNAs significantly enriched in 8 pathways (Fig. 4b). Several tumor metastasis-associated pathways such as PI3K/Akt signaling pathway and Hippo signaling pathway were included.

### PPI network construction and analysis

**Fig. 5:**
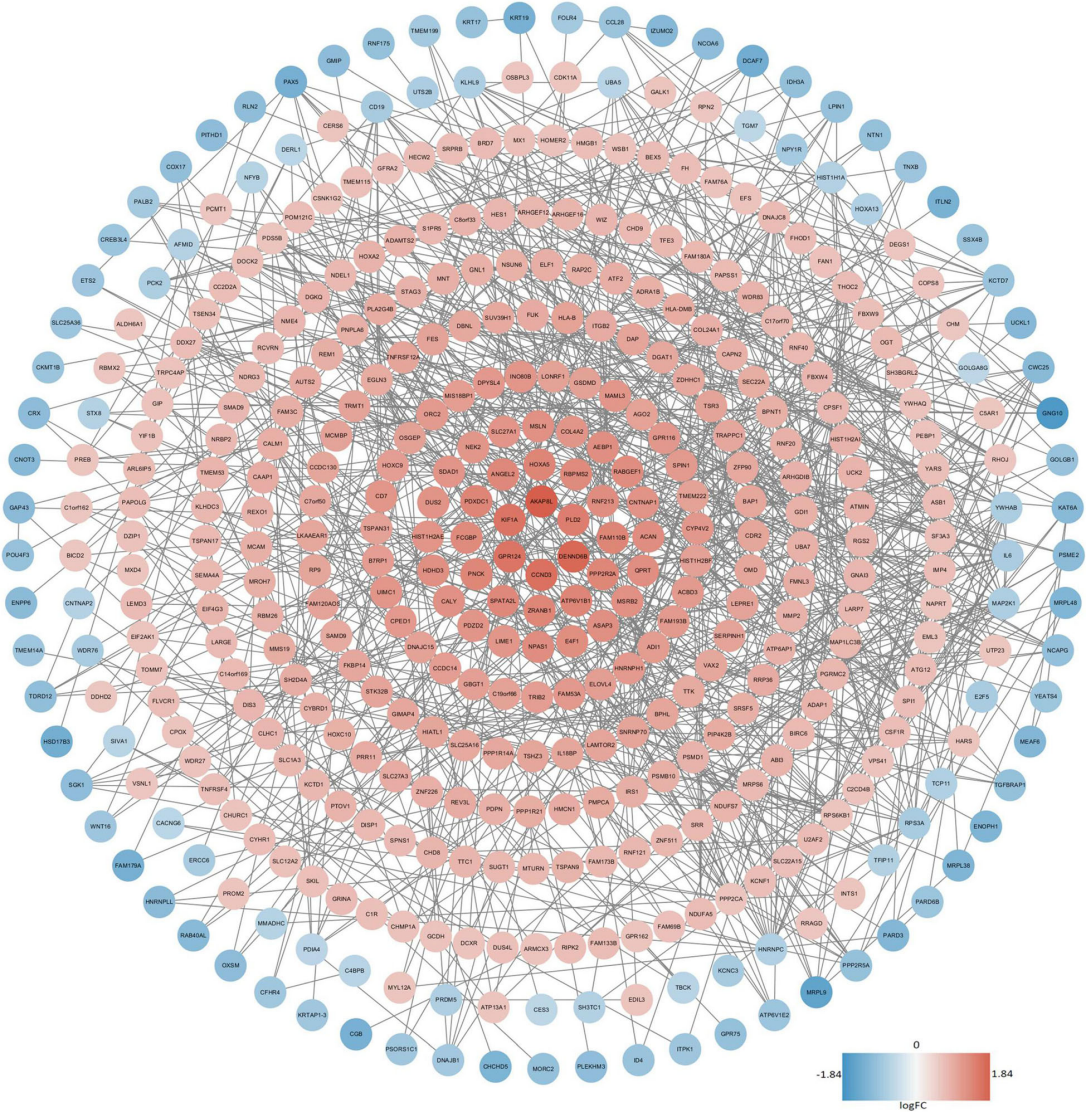
Protein-protein interaction network of DEmRNA in GSE39055. Red nodes represent up-regulated mRNAs, blue nodes represent down-regulated mRNAs.DEmRNA: differentially expressed messenger RNA;FC:fold change

The online tool STRING was used to identify the correlation of DEmRNAs and construct the PPI network. A total of 564 nodes and 900 edges were involved in the PPI network. The disconnected nodes were deleted and the PPI network was input to Cytoscape for further study (Fig. 5). The Cytoscape plug-in CytoHubba was used to screen out the top 10 hub mRNAs, which included IL6, ITGB2, PPP2CA, HNRNPC, CPSF1, YWHAB, RPS6KB1, UBA7, MAP 2 K1 and RPS3A. The KEGG pathway enrichment of these hub mRNAs was performed in DAVID and the result was shown in Table 4. It is worth noting that the tumor metastasis-associated PI3K/Akt signaling pathway was included once again.

**Table 4:** The KEGG pathway enrichment of hub DEmRNAs

KEGG pathway	count	***P*** value	mRNAs
hsa04151:PI3K-Akt signaling pathway	5	6.41E-04	IL6, MAP 2 K1, PPP2CA, YWHAB, RPS6KB1
hsa04066:HIF-1 signaling pathway	3	0.006509805	IL6, MAP 2 K1, RPS6KB1
hsa04114:Oocyte meiosis	3	0.008626936	MAP 2 K1, PPP2CA, YWHAB
hsa05161:Hepatitis B	3	0.014415179	IL6, MAP 2 K1, YWHAB
hsa04390:Hippo signaling pathway	3	0.015573394	PPP2CA, YWHAB, ITGB2
hsa05020:Prion diseases	2	0.043638711	IL6, MAP 2 K1

*KEGG* Kyoto Encyclopedia of Genes and Genomes; *DEmRNA* differentially expressed messenger RNA;*DEmiRNA* differentially expressed microRNA

### ceRNA network construction and analysis

**Fig. 6:**
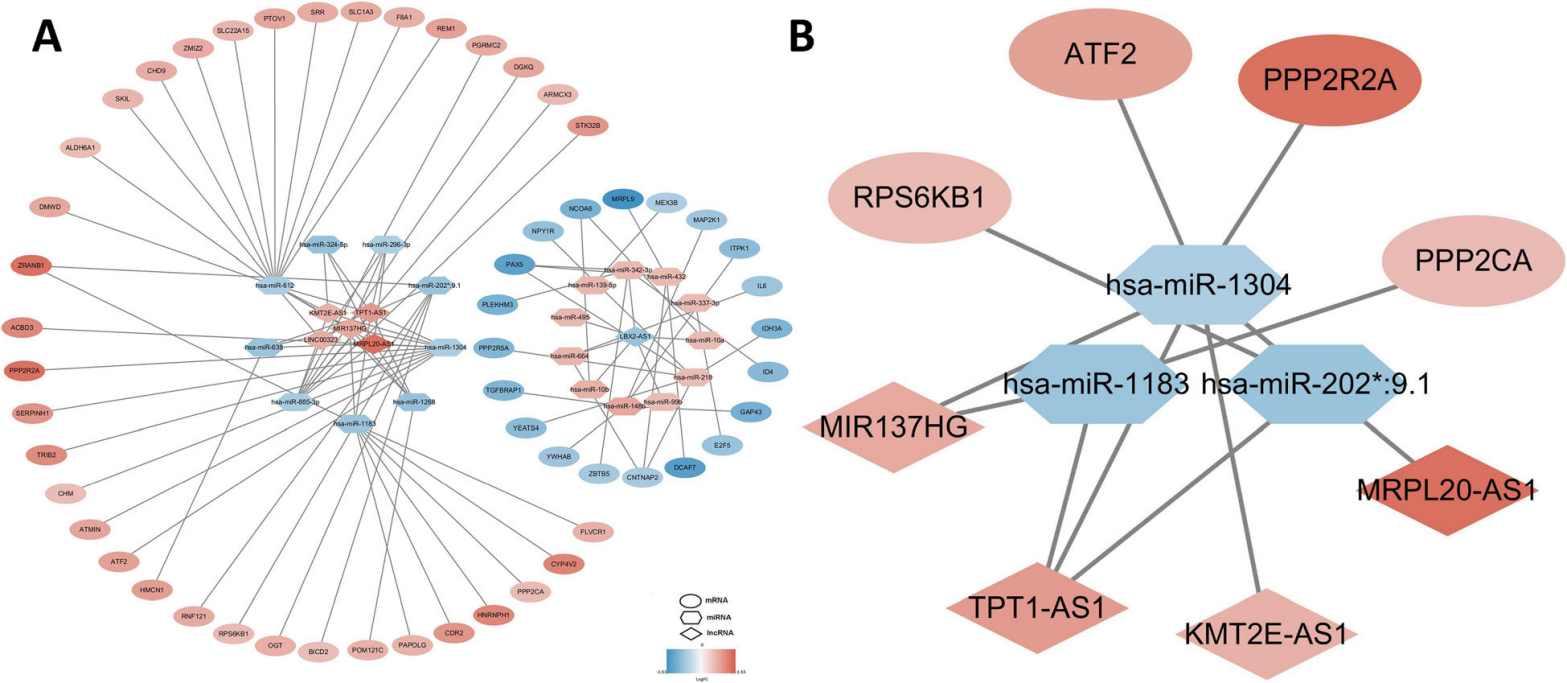
ceRNA network of DERNAs. **a** ceRNA network of all DERNAs; **b** the osteosarcoma metastasis-associated hub ceRNA module. Red nodes represent upregulated RNAs,blue nodes represent downregulated RNAs.DERNAs: differentially expressed RNA;FC:fold change;mRNA:messenger RNA;miRNA: microRNA; lncRNA: long non-coding RNA; ceRNA: competing endogenous RNA

Targetscan and miRDB were used to predict the target mRNAs of DEmiRNAs just as previously mentioned. In total, 4030 targeted mRNAs were screened out. Then these mRNAs were cross-checked with DEmRNAs in GSE39055 and the intersectional mRNAs which presented with an opposite expression trend to DEmiRNAs were selected. After that, 61 mRNA-miRNA regulatory relationships including 16 miRNAs and 55 mRNAs were obtained. The same method was used to establish the lncRNA-miRNA network through Genecode and 36 lncRNA-miRNA regulatory relationships including 6 lncRNAs and 15miRNAs were screened out. Finally, the ceRNA network which contained 6 lncRNAs,20 miRNAs and 55 mRNAs were constructed through Cytoscape (Fig. 6a).

**Fig. 7:**
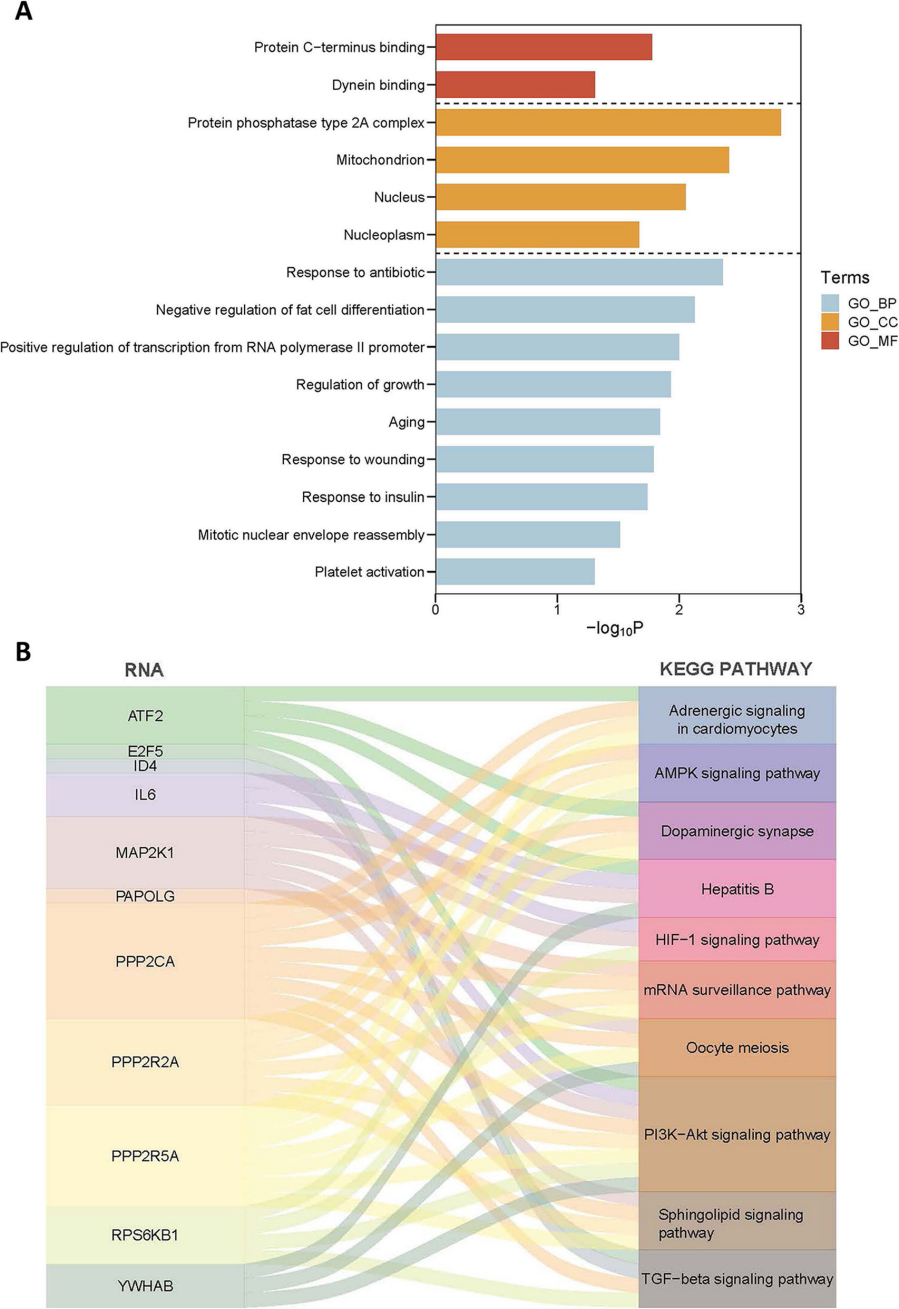
GO functional and KEGG pathway analyses of mRNAs in ceRNA network. **a** significantly enriched GO items in CC, BP and MF; **b** top 10 significantly enriched KEGG pathways. GO:gene ontology; KEGG:Kyoto Encyclopedia of Genes and Genomes; FC:fold change; DEmRNA: differentially expressed messenger RNA;BP: biology process;CC:cellular component; MF: molecular function

GO functional and KEGG pathway analyses of mRNAs in ceRNA network were conducted through DAVID. The GO functional enrichment result was shown in Fig. 7a and several biological processes such as regulation of growth, positive regulation of transcription from RNA polymerase II promoter, mitotic nuclear envelope reassembly had a significant impact on tumor metastasis [29, 30]. The outcome of KEGG pathway enrichment of mRNAs was shown in Fig. 7b and Supplementary Table 3. The tumor metastasis-associated pathways such as PI3K/Akt signaling pathway, TGF-beta signaling pathway, AMPK signaling pathway and HIF-1 signaling pathway were significantly enriched.

The top 10 hub RNAs in ceRNA network were screened out through Cytoscape plug-in CytoHubba. All hub RNAs were lncRNA and miRNA, including TPT1-AS1, hsa-miR-612, hsa-miR-1304, MIR137HG, MRPL20-AS1, hsa-miR-1183, KMT2E-AS1, hsa-miR-885-3p, hsa-miR-202*:9.1 and hsa-miR-1268. Then the KEGG pathway analysis of these miRNAs was conducted through their targeted mRNAs in ceRNA network (Table 5). The tumor metastasis-associated PI3K/Akt signaling pathway was enriched once again and we identified it as the most important osteosarcoma metastasis-associated pathway because it was the only pathway that significantly enriched in every KEGG analysis result. Based on the regulatory relationships between hub RNAs and mRNAs related to PI3K/Akt signaling pathway, the osteosarcoma metastasis-associated hub ceRNA modules were constructed (Fig. 6b).

**Table 5:** The KEGG pathway enrichment of hub miRNA targeted mRNAs in ceRNA network

KEGG pathway	count	***P*** value	mRNAs
hsa03015:mRNA surveillance pathway	3	0.008808942	PPP2CA, PAPOLG, PPP2R2A
hsa04151:PI3K-Akt signaling pathway	4	0.015271032	PPP2CA, RPS6KB1, ATF2, PPP2R2A
hsa04152:AMPK signaling pathway	3	0.015695602	PPP2CA, RPS6KB1, PPP2R2A
hsa04728:Dopaminergic synapse	3	0.016929032	PPP2CA, ATF2, PPP2R2A
hsa04261:Adrenergic signaling in cardiomyocytes	3	0.019517719	PPP2CA, ATF2, PPP2R2A

*KEGG* Kyoto Encyclopedia of Genes and Genomes; *DEmRNA* differentially expressed messenger RNA;*DEmiRNA* differentially expressed microRNA; *ceRNA* competing endogenous RNA

### Survival analysis of RNAs in osteosarcoma metastasis-associated hub ceRNA modules

**Fig. 8:**
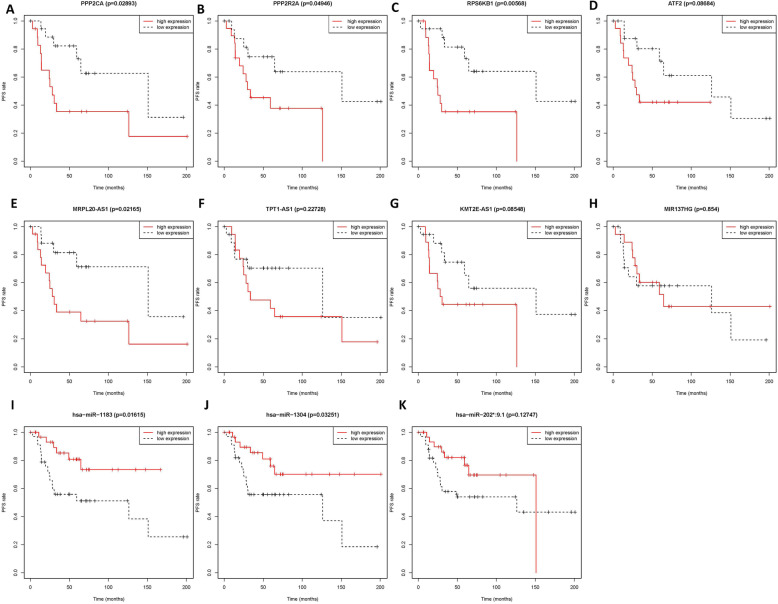
Kaplan-Meier survival curves of RNAs in hub ceRNA module. **a**-**d**: mRNA; **e**-**h**: lncRNA; **i**-**k**: miRNA.mRNA:messenger RNA; miRNA: microRNA; lncRNA: long non-coding RNA

The prognosis information of patents was download from the same datasets. The KM survival curve analysis was used to identify the relation between the expression levels of RNAs in hub ceRNA module and the progression-free survival rate (PFS) of patients (Fig. 8). The results showed that different expression levels of these RNAs had a distinct impact on patients’ PFS rate and most of them were statistically or borderline (8/11) significant, which indicated that these RNAs might be the promising prognostic biomarkers for osteosarcoma patients. What’s more, we searched these RNAs in pubmed database and the results also showed that they were correlated with osteosarcoma progression and prognosis [31–34].

## Discussion

Osteosarcoma is one of the most common bone malignant tumors and about one-fifth of patients appear clinically detectable metastases at diagnosis. The most common metastatic site of osteosarcoma is pulmonary and the 5-year survival rate of these patients is extremely low [35]. In order to improve the prognosis of patients with metastasis, the mechanism underlying metastasis was studied and first mentioned by Paget in 1889, just known as the “seed and soil” theory [36]. After that, with the development of science and technology, more theories of cancer metastasis had been proposed, such as epithelial-mesenchymal transition (EMT) enhancement, gene fusion and mutation, intravasation, enhancement of cancer stemness, increased angiogenesis and local invasion [37]. Unfortunately, the primary mechanism of osteosarcoma metastasis was still unclear and the survival time of metastatic patients was not significantly prolonged in decades.

In the last few years, the roles of lncRNA in various cancers had been widely studied. One important function of lncRNA was to bind to miRNAs and act as the miRNA sponge to regulate mRNA expression, which was correlated with tumor formation, progression and metastasis. These lncRNA-miRNA-mRNA regulatory relationships, also known as ceRNA network, had been reported in different cancers [15, 16]. However, the ceRNA network in metastatic osteosarcoma had not been revealed. Therefore, we conducted this study and looked forward to identify the key RNAs involved in the process of metastasis.

In the present study, we got 41 DEmiRNAs from GSE39040 and 22 of them were selected for further study. 564 DEmRNAs and 16 DElncRNAs were identified from GSE39055. The GO functional analysis of these aberrantly expressed RNAs showed that they were closely related to the process of cancer metastasis, such as protein binding transcription factor activity, enzyme regulator activity, protein serine/threonine/tyrosine kinase activity and so on. As for the KEGG pathway enrichment analysis, some common cancer metastasis-associated pathways were significantly enriched, including PI3K/Akt signaling pathway, Hippo signaling pathway, Ras signaling pathway and Wnt signaling pathway. The PPI network of DEmRNAs was constructed and the hub mRNAs were screened out. These hub mRNAs were closely related to the metastatic processes in various cancers. For instance, the overexpression of miR-449a directly inhibited the translation of mitogen-activated protein (MAP) kinase kinase MAP 2 K1, which decreased the activity of MEK1/ERK1/2/c-Jun pathway and suppressed the invasion and metastasis of lung cancer cells [38]. IL6, one of the proinflammatory cytokines, promoted breast cancer growth and metastasis through IL6/JAK/STAT3 loop [39]. The transcriptional co-activator Yes-associated protein (YAP) induced the expression of integrin subunit ITGB2, which increased cancer cell invasion [40]. In addition, the KEGG pathway analysis revealed that the hub mRNAs were significantly enriched in PI3K/Akt signaling pathway once again. To sum up, these analyses showed that the DERNAs obtained from two datasets played important roles in the process of cancer metastasis and the results of our study were reliable.

Next, we predicted the target mRNAs and lncRNAs of DEmiRNAs from online databases and cross-checked them with previously obtained DEmRNAs or DElncRNAs. After that, the osteosarcoma metastasis-related ceRNA network was constructed and the KEEG pathway analysis showed that the mRNAs in ceRNA network were enriched in PI3K/Akt signaling pathway once again. At the same time, the hub RNAs in ceRNA network were screened out and the miRNAs among them were enriched in PI3K/Akt signaling pathway again. Based on the results showed above, the PI3K/Akt signaling pathway was treated as the most important osteosarcoma metastasis-associated pathway because apart from it, no other pathways could be significantly enriched every time. Next, based on the PI3K/Akt signaling pathway associated hub RNAs and their targeted mRNAs, the hub osteosarcoma metastasis-associated ceRNA module was constructed. At last, the KM survival analysis showed that the vast majority of RNAs in hub ceRNA module had a significant impact on the prognosis of osteosarcoma patients.

The PI3K/AKT signaling pathway is one of the most crucial intracellular oncogenic pathways in cancers. It could be activated by G-protein-coupled receptor signaling and receptor tyrosine kinases (RTKs) such as vascular endothelial growth factor receptor (VEGFR), platelet-derived growth factor receptor (PDGFR) and insulin-like growth factor 1 receptor (IGF1R). The activation or inhibition of PI3K/AKT signaling pathway would result in a cascade of events that played important roles in pathological processes, such as oncogenesis, invasion, metastasis, angiogenesis and proliferation [41]. What’s more, almost every component of this pathway had amplifications or mutations in cancers. In the present study, we identified PI3K/AKT as the most important signaling pathway in osteosarcoma metastasis. A large number of studies had reported the same results. Zhang et al found that the Fibulin-4 could induce EMT by activating PI3K/AKT signaling pathway, which promoted osteosarcoma cell invasion and metastasis [42]. Jin et al reported that suppressing the Glycoprotein non-metastatic melanoma protein B (GPNMB) would inhibit osteosarcoma cell proliferation and metastasis via blocking PI3K/Akt/mTOR signaling pathway [43]. In the last few years, numerous studies had also revealed that ncRNAs could regulate osteosarcoma metastasis through PI3K/AKT signaling pathway. LINC00628, one of the common lncRNAs, was decreased in osteosarcoma tissues and overexpression of it would inhibit the proliferation, invasion and migration of osteosarcoma cells through PI3K/AKT signaling pathway [44]. To sum up, the PI3K/AKT signaling pathway is involved in the process of osteosarcoma metastasis and alterations in this pathway might improve the prognosis of patients.

With the development of microarray and next generation sequencing technology, numerous aberrantly expressed oncogenes were detected and most of them related to the occurrence and development of cancers. On the basis of these findings, we constructed the hub ceRNA module associated with PI3K/AKT signaling pathway, which contained three miRNAs (hsa-miR-1304, hsa-miR-202*:9.1 and hsa-miR-1183), four mRNAs (PPP2R2A, ATF2, PPP2CA and RPS6KB1) and four lncRNAs (MIR137HG, TPT1-AS1, KMT2E-AS1 and MRPL20-AS1). A number of studies found that the RNAs in our ceRNA network correlated with the proliferation, invasion and metastasis of various cancers. PPP2CA, the catalytic subunit of PP2A, was higher expressed in osteosarcoma tissues. The reduction of PPP2CA suppressed the proliferation and metastasis of osteosarcoma both in vitro and in vivo [34]. RPS6KB1 was a conserved serine/threonine kinase that served as the transcription factor in protein synthesis process. Cai et al found that the overexpression of RPS6KB1 in prostate cancer patients related to aggressive progression and poor prognosis. Meanwhile, suppressing RPS6KB1 activity by miR-195 inhibited prostate cancer invasion and migration, which provided a potential target for prostate cancer treatment [45]. MiR-1304, usually regarded as a tumor suppressor, inhibited non-small cell lung cancer (NSCLC) cell growth through suppressing heme oxygenase-1 (HO-1) and was down-regulated in NSCLC samples [46]. In a word, the genes in hub ceRNA module influenced the metastasis of cancers and might act as the therapeutic targets. However, the roles of these genes in osteosarcoma were still limited and more studies should be conducted in the future.

The KM survival analysis of our study showed that RNAs in hub ceRNA network were correlated with the patients’ survival rate, which indicated that they could serve as the predictors of prognosis. PPP2R2A, the upstream effectors of Akt/mTOR pathway, encoded an alpha isoform of the regulatory subunit B55 subfamily. Our research showed that osteosarcoma patients with higher expression of PPP2R2A had a worse prognosis, which was consistent with the study by Hein et al. [47] Zhou et al reported that the expression level of RPS6KB1 encoded protein p70S6K was negatively correlated with the progression of osteosarcoma patients. The same result was obtained in our KM survival analysis [33].

In conclusion, the present study screened out DElnRNAs, DEmiRNAs and DEmRNAs between metastatic and non-metastatic osteosarcoma patients through microarray datasets in GEO database. Importantly, the ceRNA network had been constructed and the PI3K/AKT signaling pathway was identified as the most important osteosarcoma metastasis-associated signaling pathway. The hub ceRNA module including three miRNAs (hsa-miR-1304, hsa-miR-202*:9.1 and hsa-miR-1183), four mRNAs (PPP2R2A, ATF2, PPP2CA and RPS6KB1) and four clncRNAs (MIR137HG, TPT1-AS1, KMT2E-AS1 and MRPL20-AS1) was constructed. Our KM survival analysis and other researchers’ results showed that the RNAs in hub ceRNA module might act as the novel therapeutic targets and prognostic markers for osteosarcoma patients. Based on the present study, we would continue to conduct researches in the future. It might reveal the potential mechanisms underlying osteosarcoma metastasis and improve the prognosis of osteosarcoma patients.

## Supplementary Information

**Additional file 1 Supplementary Table S1.**The clinical characteristics of patients in GSE39040 and GSE39055. Supplementary **Table S2**.The KEGG pathway enrichment of DEmiRNAs. Supplementary **Table S3**.The KEGG pathway enrichment of mRNAs in ceRNA network.

## Data Availability

The authors declare that the data supporting the findings of this study are available within the article.

## Abbreviations

ncRNA: Non-coding RNA
DERNA: Differentially expressed RNA
GEO: Gene Expression Omnibus
PPI: Protein-protein interaction
mRNA: Messenger RNA
DEmRNA: Differentially expressed messenger RNA
lncRNA: Long non-coding RNA
miRNA: microRNA
ceRNA: Competing endogenous RNA
KM: Kaplan-Meier
UTR: Untranslated region
MRE: miRNA-binding sites
TCGA: The Cancer Genome Atlas
GO: Gene Ontology
KEGG: Kyoto Encyclopedia of Genes and Genomes
Limma: Linear Models for Microarray Analysis
DAVID: Database for Annotation, Visualization and Integrated Discovery
FC: Fold change
CC: Cellular component
MF: Molecular function
BP: Biological process
STRING: Search Tool for the Retrieval of Interacting Genes database
EMT: Epithelial-mesenchymal transition
MAP: Mitogen-activated protein
YAP: Yes-associated protein
RTK: Receptor tyrosine kinase
VEGFR: Vascular endothelial growth factor receptor
PDGFR: Platelet-derived growth factor receptor
IGF1R: Insulin-like growth factor 1 receptor
GPNMB: Glycoprotein non-metastatic melanoma protein B
NSCLC: Non-small cell lung cancer
HO-1: Heme oxygenase-1
