# Supplementary material for: Development and analysis of long non-coding RNA-associated competing endogenous RNA network for osteosarcoma metastasis
Source: Hereditas. 2021 Feb 16;158:9. doi: 10.1186/s41065-021-00174-0 (PMC7887822; doi:10.1186/s41065-021-00174-0)
Supplement: Supplementary file 1 — Additional file 1 Supplementary Table S1.The clinical characteristics of patients in GSE39040 and GSE39055. Supplementary Table S2.The KEGG pathway enrichment of DEmiRNAs. Supplementary Table S3.The KEGG pathway enrichment of mRNAs in ceRNA network. [file 41065_2021_174_MOESM1_ESM.doc]

**Supplementary Table 1.The clinical** **characteristics of patients in GSE39040 and GSE39055.**

| **Variable** | **GSE39040** | **GSE39055** |
| --- | --- | --- |
| **Age** |  |  |
| <18 | 57 | 33 |
| ≥18 | 8 | 4 |
| **Gender** |  |  |
| Male | 30 | 20 |
| Female | 35 | 17 |
| **Metastasis status** |  |  |
| Non-metastasis | 24 | 18 |
| Metastasis | 41 | 19 |
| **Chemotherapy response** |  |  |
| ≥90% necrosis | 26 | 13 |
| <90% necrosis | 39 | 24 |
| **Survival status** |  |  |
| Death | 14 | 10 |
| Survive | 51 | 27 |

**Supplementary Table 2.The KEGG pathway enrichment of DEmiRNAs.**

| **KEGG pathway** | **count** | **P value** |
| --- | --- | --- |
| Proteoglycans in cancer  Pathways in cancer  TGF-beta signaling pathway  Prion diseases  Amphetamine addiction  Signaling pathways regulating pluripotency of stem cells  Hippo signaling pathway  FoxO signaling pathway  Axon guidance  ErbB signaling pathway  ECM-receptor interaction  Circadian rhythm  Wnt signaling pathway  Ubiquitin mediated proteolysis  Glioma  Ras signaling pathway  Renal cell carcinoma  Focal adhesion  Long-term potentiation  Adherens junction  Chronic myeloid leukemia  Dopaminergic synapse  AMPK signaling pathway  PI3K-Akt signaling pathway  Pancreatic cancer  Colorectal cancer  Nicotine addiction  Thyroid hormone signaling pathway  MAPK signaling pathway  Small cell lung cancer  Phosphatidylinositol signaling system  Non-small cell lung cancer  Rap1 signaling pathway  Acute myeloid leukemia  Transcriptional misregulation in cancer  Dorso-ventral axis formation  Thyroid hormone synthesis  Glycosaminoglycan biosynthesis - heparan sulfate / heparin  Arrhythmogenic right ventricular cardiomyopathy (ARVC)  Circadian entrainment  Glutamatergic synapse  Regulation of actin cytoskeleton  RNA transport  GABAergic synapse  p53 signaling pathway  Melanoma  Biotin metabolism  Morphine addiction  Endocytosis  Hedgehog signaling pathway  Adipocytokine signaling pathway  cAMP signaling pathway  Gap junction | 19  19  16  5  15  18  17  15  19  18  14  13  18  16  16  19  16  20  17  16  15  18  17  19  15  15  15  17  18  15  13  16  20  14  17  8  15  9  13  16  16  19  15  15  15  15  1  18  17  13  15  19  13 | 6.83E-09  2.14E-07  4.84E-07  7.00E-07  2.05E-06  2.66E-06  6.94E-06  1.23E-05  3.29E-05  3.71E-05  4.06E-05  7.46E-05  7.46E-05  0.000114465  0.000137822  0.000295519  0.000483551  0.00051067  0.000929452  0.001031421  0.001625923  0.002344472  0.003953676  0.004787507  0.006080106  0.006282004  0.006759995  0.007008785  0.007412814  0.007700893  0.009706796  0.010055909  0.011688184  0.011734458  0.013941102  0.014161784  0.015332381  0.015455634  0.01942802  0.01942802  0.01942802  0.01942802  0.020676336  0.022622371  0.022924132  0.024548788  0.02813204  0.02813204  0.037219734  0.042390431  0.042390431  0.042390431  0.044300469 |

KEGG:Kyoto Encyclopedia of Genes and Genomes; DEmiRNA: differentially expressed microRNA.

**Supplementary Table 3.The KEGG pathway enrichment of mRNAs in ceRNA network.**

| **KEGG pathway** | **count** | **P value** | **mRNAs** |
| --- | --- | --- | --- |
| PI3K-Akt signaling pathway  TGF-beta signaling pathway  mRNA surveillance pathway  Oocyte meiosis  Sphingolipid signaling pathway  AMPK signaling pathway  Dopaminergic synapse  Adrenergic signaling in cardiomyocytes  Hepatitis B  HIF-1 signaling pathway  Choline metabolism in cancer  Chagas disease (American trypanosomiasis)  TNF signaling pathway  Insulin resistance | 8  4  4  4  4  4  4  4  4  3  3  3  3  3 | 0.000091619  0.002607835  0.003274637  0.005724383  0.007107157  0.007608572  0.008490173  0.010428769  0.011927714  0.040320533  0.044207309  0.046604711  0.049049747  0.049875155 | IL6, MAP2K1, PPP2R5A, PPP2CA, YWHAB, RPS6KB1, ATF2, PPP2R2A  E2F5, PPP2CA, ID4, RPS6KB1  PPP2R5A, PPP2CA, PAPOLG, PPP2R2A  MAP2K1, PPP2R5A, PPP2CA, YWHAB  MAP2K1, PPP2R5A, PPP2CA, PPP2R2A  PPP2R5A, PPP2CA, RPS6KB1, PPP2R2A  PPP2R5A, PPP2CA, ATF2, PPP2R2A  PPP2R5A, PPP2CA, ATF2, PPP2R2A  IL6, MAP2K1, YWHAB, ATF2  IL6, MAP2K1, RPS6KB1  DGKQ, MAP2K1, RPS6KB1  IL6, PPP2CA, PPP2R2A  IL6, MAP2K1, ATF2  IL6, RPS6KB1, OGT |

KEGG:Kyoto Encyclopedia of Genes and Genomes; mRNA: messenger RNA;ceRNA: competing endogenous RNA.
